# Predictors of Acute Kidney Injury in Patients Hospitalized With Liver Cirrhosis: A Systematic Review and Meta-Analysis

**DOI:** 10.7759/cureus.52386

**Published:** 2024-01-16

**Authors:** Scott Nall, Hasnan Arshad, Bianca Contractor, FNU Sunina, FNU Raja, Sandipkumar S Chaudhari, Saima Batool, Adil Amin

**Affiliations:** 1 Medicine, Central Michigan University School of Medicine, Saginaw, USA; 2 Medicine, Central Park Hospital, Lahore, PAK; 3 Internal Medicine, Smt. Nathiba Hargovandas Lakhmichand (NHL) Municipal Medical College, Ahmedabad, IND; 4 Medicine, Jinnah Postgraduate Medical Centre, Karachi, PAK; 5 Pathology, MetroHealth Medical Center, Cleveland, USA; 6 Cardiothoracic Surgery, University of Alabama at Birmingham, Birmingham, USA; 7 Family Medicine, University of North Dakota School of Medicine and Health Sciences, Fargo, USA; 8 Internal Medicine, Hameed Latif Hospital, Lahore, PAK; 9 Cardiology, Pakistan Navy Ship (PNS) Shifa, Karachi, PAK

**Keywords:** systematic review and meta-analysis, incidence, predictors, liver cirrhosis, acute kidney injury

## Abstract

Acute kidney injury (AKI) frequently occurs in hospitalized individuals with liver cirrhosis and represents a significant risk factor for early in-hospital mortality, holding crucial clinical and prognostic importance. The objective of this meta-analysis was to assess the risk factors associated with AKI in hospitalized individuals with cirrhosis. This systematic review and meta-analysis was conducted in concordance with guidelines provided by the Preferred Reporting Items for Systematic Reviews and Meta-Analysis statement. Two independent researchers systematically searched major databases, including MEDLINE/PubMed, Web of Science, and EMBASE, from January 2015 until December 2023. A total of 14 studies were included in this meta-analysis, of which six were prospective, and the remaining were retrospective. Of the 9,659 cirrhosis patients in the 14 included studies, 3,968 had developed AKI with a pooled incidence of 41% (95% confidence interval = 34-47%). Our findings showed that a high Model for End-Stage Liver Disease (MELD) score, infection, high Child-Pugh-Turcotte stage score, high serum creatinine, high serum bilirubin, and low serum albumin were significantly associated with high incidence of AKI in liver cirrhosis patients. The results emphasize the importance of vigilant monitoring in cirrhosis patients to detect any indications of AKI, followed by meticulous and attentive management.

## Introduction and background

Acute kidney injury (AKI) frequently occurs in hospitalized individuals with liver cirrhosis and represents a significant risk factor for early in-hospital mortality, holding crucial clinical and prognostic importance [1]. Patients with cirrhosis exhibit a higher susceptibility to AKI compared to noncirrhotic individuals, with an estimated prevalence ranging from 20% to 50% among hospitalized cirrhosis patients [2,3]. The causes of AKI in cirrhotic patients are diverse, encompassing factors such as infections, volume depletion due to gastrointestinal (GI) bleeding or fluid losses, glomerulonephritis associated with chronic hepatitis B or C infection, hepatorenal syndrome (HRS), and nephrotoxicity [4].

Previously, AKI in cirrhosis was characterized by a 50% elevation in serum creatinine (SCr) with a final value exceeding 133 μmol/L [5]. However, due to the prevalent malnutrition and sarcopenia in individuals with liver cirrhosis [6], this definition resulted in the oversight of milder forms of AKI, lacking prompt attention and care. The current definition of AKI involves a rise in SCr by ≥0.3 mg/dL (≥26.5 μmol/L) within <48 hours, or reaching 1.5 times the baseline, occurring within the preceding seven days, in conjunction with urine output criteria of <0.5 mL/kg/hour for six hours [7]. HRS type 1 represents a distinctive form of AKI.

Numerous studies have investigated the occurrence of AKI in cirrhosis patients. The most recent systematic review, encompassing 30 studies and a total of 18,474 subjects, indicated that 29% of individuals with cirrhosis also experienced AKI [8]. However, this assessment demonstrated significant diversity, possibly arising from differences in the diagnostic criteria employed for the characterization and classification of AKI. These criteria encompassed the Acute Kidney Injury Network guidelines, International Club of Ascites 2015 criteria, and Kidney Disease Improving Global Outcomes (KDIGO) criteria, among others [9,10].

While numerous studies have investigated the occurrence and risk factors of AKI in cirrhosis patients (compensated or decompensated), there is a scarcity of pooled data from these studies. Consequently, we conducted this meta-analysis to assess the risk factors associated with AKI in hospitalized individuals with cirrhosis. This research is of significant importance as it seeks to conduct a focused meta-analysis to elucidate the risk factors linked to AKI in cirrhosis patients during hospitalization. The outcomes of this study are anticipated to provide valuable insights for identifying and understanding the factors influencing AKI in this particular patient group, thereby enhancing clinical management and care.

## Review

Methodology

We conducted this systematic review and meta-analysis in concordance with the guidelines provided by the Preferred Reporting Items for Systematic Reviews and Meta-Analysis (PRISMA) [11].

Literature Search Strategy

Two independent researchers (BC and FS) systematically searched major databases, including MEDLINE/PubMed, Web of Science, and EMBASE, from January 2015 until December 2023. The keywords used for conducting the search included “predictors,” “acute kidney injury,” and “cirrhosis,” along with relevant synonyms, incorporating relevant medical subject heading (MesH) terms and Boolean operators. Following is the search strategy for PubMed: “(“Acute Kidney Injury”[Mesh] OR “AKI”[Mesh]) AND (“Liver Cirrhosis”[Mesh] OR “Cirrhosis, Liver”[Mesh]) AND (“Predictive Factors”[Mesh] OR “Risk Factors”[Mesh]) AND (“Biomarkers”[Mesh] OR “Prognosis”[MeSH] OR “Clinical Prediction Rule”[Mesh]).” Additionally, reference lists of all included studies were manually screened to find additional studies relevant to the study topic.

Study Design and Selection Criteria

The eligibility and decision-making process for article inclusion/exclusion followed a hierarchical approach, involving a review of the title, abstract, and full text. The study selection and critical appraisal adhered to the Joanna Briggs Institute’s protocol, which offers more specific and stringent criteria for the study selection process. Only studies conducted among patients with a confirmed diagnosis of cirrhosis and assessing the risk factors of AKI were considered for inclusion. The diagnosis of cirrhosis in the selected studies utilized clinical, biochemical, radiological, or histologic examination, or a combination of these methods. We included studies regardless of the criteria used for diagnosing AKI. The predefined inclusion criteria were (1) individuals aged 18 or above and (2) observational studies (prospective and retrospective cohorts). The predefined exclusion criteria encompassed (1) studies involving animals and minors; (2) original studies comprising case reports, case series, and case-control studies; and (3) studies lacking a comparison or control group.

Data Extraction and Assessment of Quality

All articles identified through the systematic search were transferred to EndNote X9 Reference Manager (Clarivate Analytics, Philadelphia, PA, USA), where duplicates were eliminated across various online databases. Subsequently, two independent researchers conducted a thorough evaluation of the remaining articles, selecting the studies that met the predetermined inclusion criteria. The initial screening involved reviewing titles and abstracts, followed by a full-text assessment to ascertain relevance. Any discrepancies were resolved through discussion with a third researcher. Data on study characteristics, including author(s), year of publication, region/hospital, study design, sample size, and the number of patients who developed AKI, were collected. Two researchers independently conducted the methodological quality assessment of the observational studies included in this analysis using the Newcastle-Ottawa Scale. This scale evaluates biases in selection, comparability, and outcome assessment and is renowned for its content validity and interrater reliability. The assessment involved a star system, where studies were evaluated on the selection of study groups, comparability of groups, and ascertainment of exposure or outcome for case-control or cohort studies, respectively. Studies scoring eight or nine stars were categorized as having a low predicted risk of bias, while those scoring six or seven were deemed to have a predicted medium risk of bias. The investigators evaluated the risk of bias for each study and categorized it as good, fair, or poor quality based on the assigned score. This approach aids in integrating quality assessments into the interpretation of meta-analysis results.

Statistical Analysis

All statistical analyses were performed using Review Manager v.5.4 (Copenhagen: The Nordic Cochrane Centre, The Cochrane Collaboration, 2022) and STATA version 16.0 (StataCorp., College Station, TX, USA). The results of factors associated with AKI were presented as odds ratio (ORs) with 95% confidence intervals (CIs) for categorical variables and mean differences (MDs) with 95% CIs for continuous variables. We used the random-effects model to deal with variation among the study results due to differences in sample size, study setting, and population characteristics. The Higgins test (I^2^) was used to categorize heterogeneity as low (<25%), moderate (25-20%), and high (>50%) [12]. We conducted a publication bias analysis for all predictors with a minimum of 10 studies using the Egger test in STATA version 16.0. Additionally, a network meta-analysis was performed to evaluate the association between the causes of liver cirrhosis and the increased risk of developing AKI.

Results

**Figure 1:**
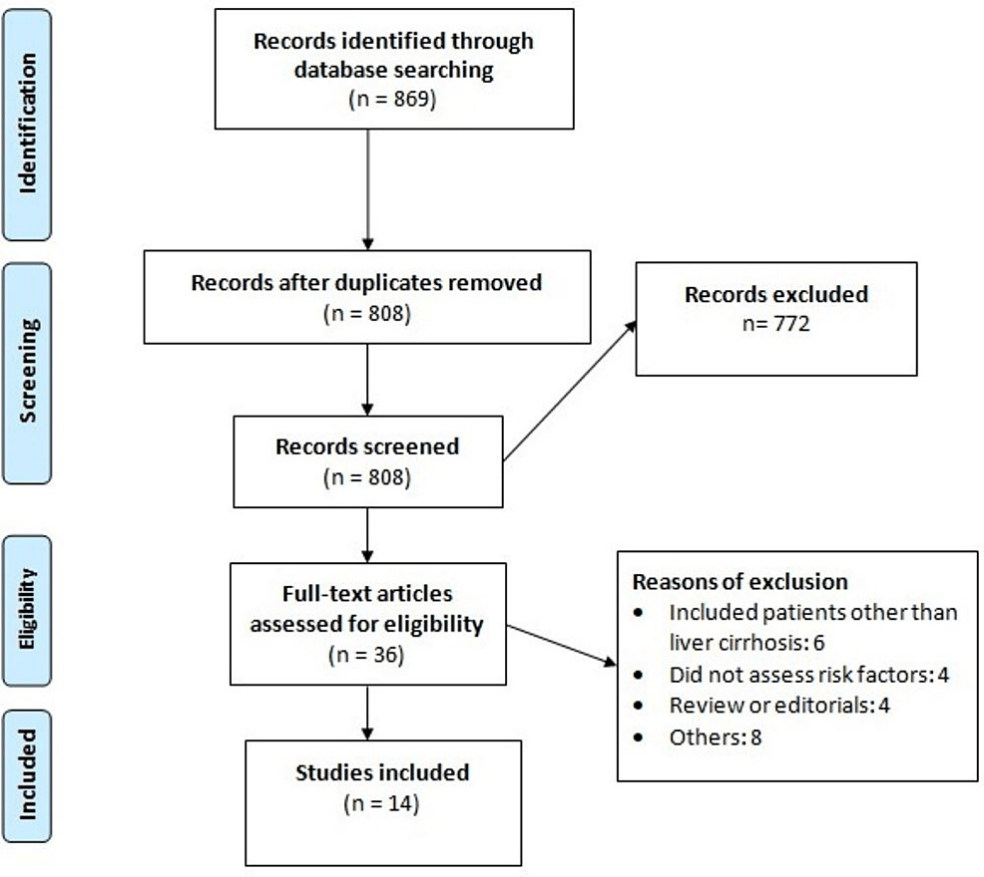
Preferred Reporting Items for Systematic Reviews and Meta-Analysis flow diagram.

Figure 1 shows the process of study selection. The systematic review of the literature uncovered a total of 869 studies. Overall, 61 studies were eliminated due to duplication, and an additional 772 studies were excluded after screening titles and abstracts. The full texts of the remaining 36 studies were thoroughly examined, leading to the exclusion of 22 studies. Finally, 14 were included in this meta-analysis, of which six were prospective, and the remaining were retrospective. The studies were conducted in China. Table 1 presents the characteristics of the included studies. Table 2 presents the quality assessment of the included studies.

**Table 1:** Characteristics of the studies included in this meta-analysis. AKI: acute kidney injury

Study ID	Year	Region	Study design	Sample size	AKI (n)	Summary
Biyik et al. [13]	2016	Turkey	Retrospective	277	169	The occurrence of AKI among cirrhotic patients was 39%, with an associated overall hospital mortality rate of 15.5%
Bucsics et al. [14]	2015	Austria	Retrospective	239	75	The median survival in patients with AKI was significantly lower compared to non-AKI patients (768 days; 95% confidence interval (CI) = 331–1,205 days)
Duah et al. [15]	2022	Ghana	Prospective	179	50	The incidence of AKI was 27.9%. The primary triggers for AKI included infections, refractory ascites, alkaline phosphatase, and Model for End-Stage Liver Disease scores
Gameiro et al. [16]	2018	Portugal	Retrospective	186	52	A total of 52 patients (28%) experienced AKI. Elevated baseline serum creatinine levels (p < 0.001), increased severity of liver disease assessed using the modified Model of End-stage Liver Disease score, and a higher neutrophil-to-lymphocyte ratio were identified as independent factors linked to the development of AKI
Hsieh et al. [17]	2017	Taiwan	Retrospective	113	46	The Child-Pugh score, systemic blood pressure upon admission, and the quantity of blood units transfused before endoscopy were identified as independent predictors of AKI
Huelin et al. [18]	2017	Spain, Italy	Prospective	547	290	The predictors for stage 1B AKI were hepatorenal syndrome and acute tubular necrosis, while hypovolemia emerged as the primary cause for stage 1A AKI
Jaques et al. [19]	2019	Switzerland	Prospective	105	55	Serum creatinine, cystatin C, and neutrophil gelatinase-associated lipocalin are independent predictors of AKI
Khatua et al. [20]	2020	India	Prospective	576	315	In contrast to individuals without AKI, those with AKI exhibited elevated white blood cell count, increased levels of serum urea and creatinine, higher Child-Turcotte-Pugh scores, and higher Model of End-Stage Liver Disease scores
Kogiso et al. [21]	2021	Japan	Retrospective	199	46	Hepatic encephalopathy significantly increased AKI risk, while higher albumin levels reduced it. Treatment with PPI/H2 blockers or kanamycin/rifaximin was associated with a reduced risk of AKI development
Gessolo Lins et al. [22]	2018	Brazil	Retrospective	258	139	The overall occurrence of AKI among cirrhotic patients reached 53.9%, with an associated overall hospital mortality rate of 28.4%
Lasheen et al. [23]	2017	Egypt	Prospective	900	393	Hemoglobin and serum albumin were notably lower in AKI patients. Serum creatinine correlated positively with leukocyte count, C-reactive protein (CRP), and international normalized ratio. Conversely, it negatively correlated with hemoglobin and albumin. CRP emerged as the most independent risk factor
Patidar et al. [24]	2019	United States	Prospective	397	59	In this study, the End-Stage Liver Disease score, admission creatinine, international normalized ratio, and white blood cell count were independently associated with AKI
Tandon et al. [25]	2016	Canada	Retrospective	4,733	1,850	Examining 4,733 participants, AKI was linked to higher 30-day mortality (43.9% vs. 8.5%; p < 0.001), escalating with severity
Tian et al. [26]	2023	China	Retrospective	950	429	Mechanical ventilation, vasopressor use, international normalized ratio, bilirubin, Charlson comorbidity index, prothrombin time, estimated glomerular filtration rate, partial thromboplastin time, and heart rate were identified as predictors of AKI

**Table 2:** Quality of the included studies using the Newcastle-Ottawa scale.

Study ID	Selection	Comparability	Outcome	Overall
Biyik et al. [13]	2	3	3	Good
Bucsics et al. [14]	2	3	3	Good
Duah et al. [15]	3	3	3	Good
Gameiro et al. [16]	2	2	2	Fair
Hsieh et al. [17]	2	3	3	Good
Huelin et al. [18]	3	3	3	Good
Jaques et al. [19]	3	3	3	Good
Khatua et al. [20]	2	2	3	Fair
Kogiso et al. [21]	2	3	3	Good
Gessolo Lins et al. [22]	2	3	3	Good
Lasheen et al. [23]	2	3	4	Good
Patidar et al. [24]	3	3	3	Good
Tandon et al. [25]	2	2	3	Fair
Tian et al. [26]	2	2	3	Fair

Incidence of Acute Kidney Injury

Of the 9,659 cirrhosis patients in the 14 included studies, 3,968 had developed AKI with a pooled incidence of 41% (95% CI = 34-47%). The pooled data exhibited high heterogeneity, indicated by an I^2^ value of 97.1%. Subgroup analysis was done by including only prospective studies and pooled incidence of AKI in prospective studies was 41% (95% CI = 27-55%).

Risk Factors Associated With the Development of Acute Kidney Injury

We assessed the impact of 11 variables at admission among 14 studies as predictors for the development of AKI among liver cirrhosis patients. Of these 11 factors, infection, a high Model for End-Stage Liver Disease (MELD) score, a high Child-Pugh-Turcotte score, high SCr, high serum bilirubin, and low serum albumin were significantly associated with a high incidence of AKI in patients with liver cirrhosis, as shown in Table 3. Other factors, including age, gender, diabetes, hypertension, and hemoglobin levels, were not significantly associated with the development of AKI, as shown in Table 3. We conducted a publication bias assessment for age, gender, and creatinine level, and no evidence of publication bias was observed, as indicated by p-values greater than 0.05.

**Table 3:** Risk factors associated with the development of AKI. ^: presented as MD (95% CI); *: significant at p-value <0.05. OR: odds ratio; MD: mean difference; CI: confidence interval; MELD: Model for End-Stage Liver Disease; AKI: acute kidney injury

Variables	Number of studies	OR (95% CI)	I^2^
Age^	11	0.92 (-0.1 to 1.95	44%
Gender (male)	13	1.15 (0.90 to 1.46)	68%
Diabetes	7	1.14 (0.95 to 1.37)	15%
Hypertension	5	1.23 (0.90 to 1.67)	0%
Hemoglobin^	5	-0.04 (-0.24 to 0.17)	0%
Infection	4	1.87 (1.15 to 2.82)	11%
Creatinine^	11	0.57 (0.20 to 0.95)*	96%
Bilirubin^	9	0.24 (0.12 to 0.36)*	48%
MELD^	9	0.82 (0.45 to 1.20)*	95%
Albumin^	7	-0.31 (-0.45 to -0.17)*	53%
Child-Pugh-Turcotte score^	5	0.48 (0.05 to 0.90)*	86%

**Figure 2:**
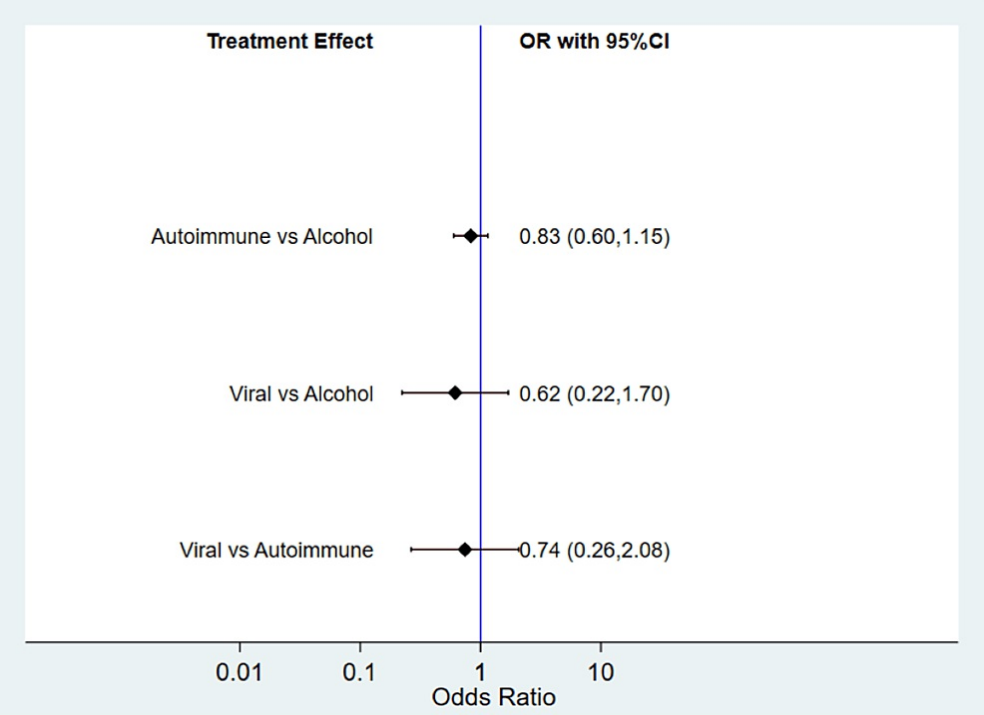
Comparison of AKI incidence in different etiologies of cirrhosis. The point estimate shows pooled OR represented by a square, and the line’s length indicates the width of the CI. AKI: acute kidney injury; OR: odds ratio; CI: confidence interval

In our study, we conducted a comparative analysis to assess the risk of AKI across different etiologies of cirrhosis, namely, autoimmune, alcoholic, and viral. The findings of this comparison are visually presented in Figure 2. The pooled analysis of the data revealed that there was no statistically significant difference in the risk of developing AKI among individuals with autoimmune, alcoholic, and viral cirrhosis. This suggests that, based on our study, the likelihood of experiencing AKI does not significantly vary across these specific etiologies of cirrhosis.

Discussion

A major aim of this meta-analysis is the investigation of risk factors for developing AKI in patients with liver cirrhosis. Our findings show that a high MELD score, infection, high Child-Pugh-Turcotte stage, high SCr, high serum bilirubin, and low serum albumin were significantly associated with a high incidence of AKI in liver cirrhosis patients.

The MELD score is widely utilized globally to assess patient outcomes and survival in individuals with cirrhosis [27]. This scoring system offers an estimation of the severity of liver disease. The results of this meta-analysis indicate that as the MELD score increases, there is a corresponding rise in the risk of developing AKI. The Child-Pugh-Turcotte score, often referred to simply as the Child-Pugh score or stage, is a clinical tool used to assess the severity of liver disease and predict the prognosis of individuals with cirrhosis [28]. The Child-Pugh score is a recognized prognostic tool for individuals with cirrhosis. While it does not incorporate a specific marker for renal function, the score correlates directly with factors such as angiotensin and aldosterone blood levels, cardiac output, and portal venous pressure gradient. These parameters serve as objective indicators of circulatory dysfunction in cirrhotic patients. Additionally, the Child-Pugh score shows an inverse correlation with renal perfusion [29].

Our study demonstrates high Child-Pugh score is associated with an increased risk of AKI. These findings are consistent with those of a review conducted by Tariq et al., reinforcing the association between higher MELD scores, higher Child-Pugh scores, and an increased likelihood of AKI development [8]. These associations are valuable in clinical contexts, especially for healthcare professionals managing patients with liver disease, as they underscore the importance of monitoring and managing kidney function, particularly in individuals with more advanced liver disease.

This meta-analysis showed that high bilirubin is one of the risk factors for AKI. Different studies have also identified bilirubin as a risk factor for AKI [30,31]. Elevated bilirubin levels can lead to oxidative stress on renal tubular cells, trigger apoptosis, and exacerbate renal ischemia-reperfusion injury, thereby playing a role in the onset of AKI [32].

Similar to the findings of Tariq et al. [8], our study also found a significant association of sepsis or septic shock with AKI in cirrhosis patients. This association can be attributed to the complex interplay between systemic inflammation, compromised immune function in cirrhosis, and the potential for bacterial infections to exacerbate existing liver and kidney dysfunction [33]. Sepsis, a severe immune response to infection, can contribute to hemodynamic changes and organ dysfunction, including renal impairment, in individuals with cirrhosis. The compromised physiological state in cirrhotic patients may make them more susceptible to the detrimental effects of sepsis on kidney function [34].

Limitations

The studies included in our meta-analysis exhibited heterogeneity in terms of study design, patient demographics, and cirrhosis status, leading to notable heterogeneity. Utilizing pooled data derived from individual patient information across these studies could potentially address this limitation, offering more uniform data on the incidence of AKI, its impact on outcomes, and variables predicting AKI. Additionally, the scarcity of data within the included studies on mortality rates in subgroups with distinct AKI stages precluded us from conducting a comprehensive pooled analysis of mortality based on AKI severity.

## Conclusions

Our meta-analysis, comprising 14 studies involving 3,968 cirrhosis patients, unveiled a pooled incidence of 41% for AKI. Notably, risk factors significantly associated with AKI included elevated MELD score, Child-Pugh-Turcotte stage, infection, SCr, bilirubin levels, and low serum albumin. Further prospective research should strive to enhance data homogeneity and explore additional facets of this complex relationship for a more comprehensive understanding. The results emphasize the importance of vigilant monitoring in cirrhosis patients to detect any indications of AKI, followed by meticulous and attentive management.
